# Supplementary material for: Complexome profiling of the Chlamydomonas psb28 mutant reveals TEF5 as an early PSII assembly factor
Source: Plant Cell. 2025 Mar 17;37(6):koaf055. doi: 10.1093/plcell/koaf055 (PMC12164590; doi:10.1093/plcell/koaf055)
Supplement: koaf055_Supplementary_Data [file koaf055_supplementary_data.zip › psb28_interactive.zip!/plots/intensity/qualityPlots/peptides/CAX1_Cre12.g519500.t1.2.html]

### Cre12.g519500.t1.2

Trivial name: CAX1  
SeqCoverage [median]: 5.10  
Mass: 47.46 kDa  
  
 

| Sequence | MissedCleavage | Mass [Da] | wtIntensity | muIntensity | Score |
| --- | --- | --- | --- | --- | --- |
| GSILSVAPK | 0 | 870.52 | 670.24 | 0.00 | 95.53 |
| NSITGDR | 0 | 761.37 | 0.00 | 3445.93 | 97.80 |
| THSELFK | 0 | 860.44 | 0.00 | 539.96 | 74.42 |
|  |  |  | 670.2 | 3985.9 |  |
